# Assessing electronic immunization registries: the Pan American Health Organization experience

**DOI:** 10.26633/RPSP.2019.28

**Published:** 2019-03-15

**Authors:** M. Carolina Danovaro-Holliday, Marcela P. Contreras, Dalys Pinto, Ida Berenice Molina-Aguilera, Diana Miranda, Odalys García, Martha Velandia-Gonzalez

**Affiliations:** 1 World Health Organization Department of Immunization, Vaccines and Biologicals Expanded Programme on Immunization Strategic information Group Geneva Switzerland Strategic information Group, Expanded Programme on Immunization, Department of Immunization, Vaccines and Biologicals, World Health Organization, Geneva, Switzerland.; 2 Comprehensive Family Immunization Unit Comprehensive Family Immunization Unit Pan American Health Organization Washington, D.C. United States of America Pan American Health Organization, Comprehensive Family Immunization Unit, Washington, D.C., United States of America.; 3 Programa Ampliado de Inmunización Programa Ampliado de Inmunización Ministerio de Salud de la República de Panamá Panamá Panamá Ministerio de Salud de la República de Panamá, Programa Ampliado de Inmunización, Panamá, Panamá.; 4 Centro Nacional de Biológicos Centro Nacional de Biológicos Secretaría de Salud de Honduras TegucigalpaFrancisco Morazán Honduras Secretaría de Salud de Honduras, Centro Nacional de Biológicos, Tegucigalpa, Francisco Morazán, Honduras.; 5 Región de San Miguelito - San Miguelito Salud Región de San Miguelito - San Miguelito Salud Panamá Panamá Región de San Miguelito - San Miguelito Salud, Panamá, Panamá.; 6 Pan American Health Organization Pan American Health Organization Tegucigalpa Honduras Pan American Health Organization, Tegucigalpa, Honduras.

**Keywords:** Immunization, electronic health records, information systems, Latin America, Caribbean Region, Inmunización, registros electrónicos de salud, sistemas de información, América Latina, Región del Caribe, Imunização, registros eletrônicos de saúde, sistemas de informação, América Latina, Região do Caribe

## Abstract

**Objective.:**

To develop a methodology to assess electronic immunization registries (EIRs) in low- and middle-income countries (LMICs) in Latin America and the Caribbean.

**Methods.:**

A team from the Immunization Unit at the Pan American Health Organization (PAHO) reviewed existing methodologies to evaluate health information systems, particularly the Performance of Routine Information System Management (PRISM) framework and methodologies used to assess information systems. In 2014, the PAHO team convened a small working group to develop an evaluation approach to be added to the existing World Health Organization immunization data quality self-assessment (DQS) tool. The resulting DQS with an added EIR component was named “DQS Plus.” The DQS Plus methodology was used in Panama in May 2014 and in Honduras in November 2015.

**Results.:**

The DQS Plus tool proved feasible and easy to implement in Panama and Honduras, including by not adding much time or resources to those needed for a usual DQS. The information obtained from the DQS Plus assessment was practical and helped provide health authorities with recommendations to update and improve their EIR, strengthen the use of the registry, and enhance the data the assessment produced, at all levels of the health system. These recommendations are currently being implemented in the two countries.

**Conclusions.:**

The DQS Plus proved to be a practical and useful approach for assessing an EIR in an LMIC and generating actionable recommendations. Further work on defining operational and related EIR functional standards in LMICs will help develop an improved EIR assessment tool for Latin America and the Caribbean, and potentially elsewhere.

## RESUMEN

**Objetivo.:**

Formular una metodología para evaluar los registros de inmunización electrónicos en los países de ingresos bajos y medianos de América Latina y el Caribe.

**Métodos.:**

Un equipo de la Unidad de Inmunizaciones de la Organización Panamericana de la Salud (OPS) examinó las metodologías existentes para evaluar los sistemas de información de salud, en particular el marco *Performance of Routine Information System Management* (PRISM), que analiza la gestión de la información sistemática, así como otras metodologías empleadas para evaluar los sistemas de información. En el 2014, el equipo de la OPS reunió a un pequeño grupo de trabajo para idear una estrategia de evaluación que se le agregaría a la herramienta existente de la Organización Mundial de la Salud para autoevaluación de la calidad de los datos (DQS) relativos a la inmunización. La herramienta DQS resultante, con un componente añadido de registros de inmunización electrónicos, se denominó “DQS Plus”. La metodología de DQS Plus se usó en Panamá en mayo del 2014 y en Honduras en noviembre del 2015.

**Resultados.:**

Se demostró que la herramienta DQS Plus fue factible y fácil de aplicar en Panamá y Honduras, entre otras cosas por no requerir mucho tiempo ni recursos adicionales a los necesarios para la DQS ordinaria. La información obtenida mediante la evaluación con DQS Plus fue práctica y contribuyó a proporcionar a las autoridades sanitarias las recomendaciones para actualizar y mejorar sus registros de inmunización electrónicos, fortalecer el uso del registro y mejorar los datos que arrojó la evaluación, a todos los niveles del sistema de salud. En la actualidad están poniéndose en práctica dichas recomendaciones en los dos países.

**Conclusiones.:**

Se demostró que DQS Plus es una estrategia práctica y útil para evaluar un registro de inmunización electrónico en los países de ingresos bajos y medianos y generar recomendaciones aplicables. El trabajo ulterior para definir las normas operativas y funcionales de los registros de inmunización electrónicos en los países de ingresos bajos y medianos contribuirá a crear una mejor herramienta de evaluación de dichos registros en América Latina y el Caribe, y posiblemente en otros sitios.

## RESUMO

**Objetivo.:**

Elaborar uma metodologia para avaliar os registros eletrônicos de vacinação em países de baixa e média renda na América Latina e no Caribe.

**Métodos.:**

Uma equipe da Unidade de Imunização da Organização Pan-Americana da Saúde (OPAS) analisou as metodologias existentes para avaliação dos sistemas de informação em saúde, em particular a estrutura de Desempenho da Gestão Rotineira dos Sistemas de Informação (PRISM) e as metodologias usadas para avaliação de sistemas de informação. Em 2014, a equipe da OPAS formou um pequeno grupo de trabalho com a incumbência de desenvolver um método de avaliação a ser integrado à ferramenta existente de autoavaliação da qualidade dos dados de imunização (DQS) da Organização Mundial da Saúde (OMS). A ferramenta DQS com o novo componente de registros eletrônicos de vacinação foi denominada “DQS Plus”. A metodologia DQS Plus foi empregada no Panamá, em maio de 2014, e em Honduras, em novembro de 2015.

**Resultados.:**

A ferramenta DQS Plus provou ser viável e fácil de ser implementada no Panamá e em Honduras, principalmente por não despender mais tempo ou recursos aos já necessários com a habitual ferramenta DQS. As informações obtidas na avaliação com a ferramenta DQS Plus foram práticas e contribuíram com recomendações às autoridades sanitárias de atualizar e melhorar os registros eletrônicos de vacinação, reforçar o uso do registro e aprimorar os dados produzidos com a avaliação em todos os níveis do sistema de saúde. Essas recomendações estão atualmente em fase de implementação nos dois países.

**Conclusões.:**

A ferramenta DQS Plus é comprovadamente um método prático e útil para avaliar os registros eletrônicos de vacinação em países de baixa e média renda e gerar recomendações executáveis. Outros estudos com o objetivo de definir os padrões operacionais e funcionais relacionados aos registros eletrônicos de vacinação nos países de baixa e média renda devem contribuir para o desenvolvimento de uma versão aprimorada da ferramenta de avaliação de registros eletrônicos de vacinação para a América Latina e o Caribe e possivelmente para outras regiões.

Electronic immunization registries (EIRs) have been defined as confidential, computerized, population-based systems that collect and consolidate vaccination data from vaccination providers for better immunization strategies (1, 2). Evidence suggests that EIRs can help increase vaccination coverage. This is mainly related to their ability to facilitate several things: a) individual follow-up of vaccination status; b) tracking defaulters (persons who are overdue for a vaccine dose) and automatically generating messages for recalls (to alert persons about overdue doses) and for reminders (to alert persons about upcoming doses); c) provider assessment and feedback; and d) vaccination clinical decision support. This is in addition to facilitating the informed management of immunization programs (1-4).

Latin American and Caribbean (LAC) countries are quickly adopting EIRs (3). The main factor associated with this move is the increased complexity of the Expanded Program on Immunization (EPI). The EPI was set up by the World Health Organization (WHO) in 1974 and adopted by the Pan American Health Organization (PAHO) in 1977, with all the PAHO Member States endorsing the EPI approach for vaccination efforts in in their countries. In the Americas, the EPI effort has evolved from 6 vaccines against 6 diseases in 1977 to at least 20 vaccine doses against 13 diseases in 2017, with costs having multiplied 10- to 15-fold over the last decade (5).

Other factors contributing to EIR development and implementation are the increased availability of information and communications technologies (6), geographic information systems, and connectivity (7-9). Since 2009, PAHO’s Technical Advisory Group on Vaccine-preventable Diseases (TAG) has recommended that countries using EIRs share experiences and lessons learned. The TAG has also stressed the importance of EIRs meeting the needs of local levels, as well as the value of systematic monitoring of EIR implementation and of evaluating country experiences so as to continue fostering the exchange of experiences, lessons learned, and good practices (10).

The immunization data quality self-assessment (DQS) tool was developed by the WHO to help countries diagnose problems and make improvements in collecting and using immunization data at the national, provincial, and district (municipal) levels (11). Since 2005, the DQS has been used with PAHO support in 27 LAC countries (either in a stand-alone process or integrated into an EPI review (12)), and by the national immunization programs in other LAC countries (information on exactly how many not available) (10, 13). Since 2006, PAHO’s DQS has included a data and information system desk review that is similar to the one described by Scott et al. (14). Furthermore, LAC countries include data quality and information systems as a separate EPI component in annual plans of action and multiyear strategic plans, as PAHO-supported DQSs always result in an improvement plan. While the DQS has been used in LAC to evaluate the data produced by an EIR (15-18), the DQS does not include a module to assess the functionality or other characteristics of an EIR.

This article describes the steps that the Immunization Unit at PAHO took to add a module for EIR assessment to its DQS methodology, its use in Panama in May 2014 and in Honduras in November 2015, and the way forward with this new evaluation approach.

## MATERIALS AND METHODS

In 2014, a member of PAHO’s Immunization Unit conducted a literature review on available methodologies and tools for EIR evaluation, using the terms “immunization information system” (IIS) and “immunization registry” (immuni* AND information AND system*) OR (electronic AND immuni* AND regist*). One article describing the assessment of the data produced by the EIR in Uruguay was identified (15). Other articles described registries from developed countries (1, 2, 19), and only two papers dealt with assessing EIRs (20, 21). We did not identify any articles proposing methodologies to assess EIRs in low- or middle-income countries (LMICs).

In addition to the published papers mentioned above, PAHO team members reviewed the report from the Uruguay DQS 2006 (16) and reports of assessments conducted in countries that were in the process of implementing an EIR: Belize DQS 2011 (17) and Colombia EPI Review 2012 (18). The goal was to evaluate the specific EIR questions added and the findings. These assessments had added questions to get a sense of EIR users’ perceptions. In Colombia, the 2012 assessment looked at the data produced by two existing subnational EIRs, one from the city of Bogotá and one from the department of Antioquia (except the city of Medellín), comparing data from those EIRs with the data from the national aggregate data collection system. Finally, we reviewed the Performance of Routine Information System Management (PRISM) framework methodology (22), as it is a well-known approach for assessing health information systems and is widely used in LMICs.

In April 2014, we convened a small ad hoc working group with persons from Colombia’s national Immunization Program; the Immunization Program of the Health Secretariat of Bogotá; the Training Programs in Epidemiology and Public Health Interventions Network (TEPHINET) in Central America (which has been collaborating with the U.S. Centers for Disease Control and Prevention (CDC) and PAHO); and a representative from the Expanded Program on Immunization of the World Health Organization headquarters. The members of this ad hoc working group were familiar with the DQS, the PRISM methodology, and EIR development and implementation.

Using PAHO’s working definition of an “ideal” EIR, the group discussed the dimensions that could be assessed, in addition to the data produced by the EIR (3, 23, 24). At the time, this definition incorporated these elements:

- inclusion of all persons at birth, or as early as possible
- unique identification (ID): national ID, or biometrics or birth registration, or a unique combination of variables such as names, date of birth, and/or place of birth, parental names or IDs, etc.
- information about each person, including information on geographical area of residence
- information about the vaccines given, dates, and provider
- aggregation of data by geographical level, as required
- timely individualized follow-up of vaccination schedule
- data entry as close to vaccination as possible (time and place)
- data security and protection of patient confidentiality

The working group decided to use seven dimensions to describe the EIR in a new DQS tool (the seven are listed in Table 1, which appears later in this article). The elements in these dimensions were to be assessed by observation; by review of the software, norms, and manuals; and from the responses to questions that were added to the DQS questionnaires for the national, subnational, and local levels, including users’ perceptions at the local level (EPI nurses and data entry clerks). We nicknamed this new tool “DQS Plus.” The Immunization team from the Health Secretariat of Bogotá pilot tested some of the proposed questions that were to be part of the DQS Plus during its routine supervision activities, before the full DQS Plus was conducted in Panama.

## RESULTS

The implementation of the DQS Plus exercises in both Panama and Honduras used the same timeline, assessment team composition, and process for developing recommendations and a plan of action as do any other DQS assessments supported by PAHO.

It took a pre-assessment visit of two to three days for a staff member from the PAHO Immunization Unit to collect information and adapt the DQS Plus tools (forms) to the country. The objectives of this pre-assessment visit were to get enough information about the country’s immunization program and information system to adapt the DQS Plus tools, to list the key informants (or entities) that would be interviewed during the DQS Plus, and to preselect the regions to be visited, so that the logistics could be in place for the actual DQS Plus exercise. It was also the start of the data/information system desk review. In this pre-assessment visit, the PAHO person leading the DQS Plus was accompanied by a nationally appointed EPI focal point and the immunization focal point from the PAHO country office.

The actual DQS Plus took six to seven days. This included two days for preparation and training, two to three days of field work, a weekend for data analysis and to draft a report and a presentation, and one day to share the main findings, recommendations, and proposed plan of action for improvement with national (and in some cases subnational) immunization stakeholders. The DQS Plus assessment teams consisted of two or three persons from the country’s EPI and/or statistics department of the ministry of health (from national or subnational levels, but not the same jurisdiction being assessed), plus an external person (usually from PAHO or from another country that had done a DQS or was planning one). A team member from the PAHO Immunization Unit stayed at the national level to conduct the desk review.

The development of recommendations used the same process as any PAHO-led DQS and EPI evaluations (12). In this process, members of the DQS Plus assessment team, including the national participants, discuss the findings and propose activities to address each problem identified, taking into consideration the national context and the specific needs of the country. These preliminary recommendations are presented to national authorities on the last day of the evaluation mission for consensus and commitment to implementation and follow-up. These recommendations are also turned into actions and added to EPI plans of action.

Below we present brief summaries of the DQS Plus implementation and findings in Panama and Honduras. (The two DQS Plus reports can be obtained upon request to PAHO by emailing immunization@paho.org and using “DQS Plus” in the email subject line.)

### Panama

In 2013, Panama requested PAHO’s support to conduct a DQS to improve the quality of the immunization coverage data being reported, as well as the timeliness of reporting. Given that Panama uses an information system with an EIR module, the country requested that this information system be specifically evaluated with the DQS, thus the need to add the EIR module described above. This DQS Plus was conducted from 22 to 30 May 2014.

#### The EIR system in Panama.

The immunization information system used in Panama (“PAI Software”) was developed in 2006-2007 (3, 25). (PAI is the abbreviation for the Spanish-language term *Programa Ampliado de Inmunización* (Expanded Program on Immunization).) The PAI Software has a module to capture aggregate data for doses administered and a module to register data person by person (EIR module). PAI Software was developed using Visual FoxPro 6.0, a now-discontinued Microsoft programming language. Since its creation, PAI Software has been adapted to incorporate new vaccines; the number of vaccines in the immunization schedule totaled 14 at the time of the DQS Plus. PAI Software is not interoperable with other information systems used by Panama’s Ministry of Health or the Social Security system, or with the information system used by Panama’s national Expanded Program on Immunization (EPI) to manage vaccine logistics and stock. PAI Software includes all vaccines used by Panama’s national EPI and can produce reports of vaccines administered, according to age, sex, dose, district of residence, and health facility. It does not produce coverage reports, as the denominator to calculate coverage comes from a national birth registry.

#### Data flow.

Each health facility (whether public, private, or Social Security) uses a daily registry form to catalog all vaccines administered at the facility, during outreach, or in school. In most cases, the data from this daily registry form is entered, person by person, into PAI Software by a clerk in the statistics office of the health facility. In other cases, the paper daily forms are sent to the health region to be entered at that level. In contrast, most health facilities from the private sector and Social Security manually aggregate data from the daily registry and enter it in the monthly report, which is also paper-based. This monthly report is submitted to the health region for data entry into the PAI Software aggregation module. The national EPI receives a file with vaccination data from each health region via e-mail. This file contains some person-by-person data, and some aggregated data.

#### Main results.

**FIGURE 1:**
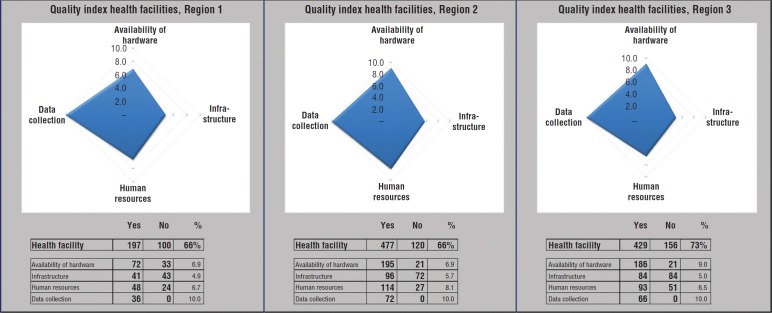
Sample DQS Plus results, with health facility average quality index, by region, Panama, 2014^a^

***Source:*** DQS Plus results from Panama in 2014.

^a^ Each item (availability of hardware, infrastructure, human resources, and data collection) represents a section included in the DQS Plus data quality questionnaire/checklist. (For Panama, Internet access was not assessed since the country’s PAI Software does not need Internet access for its functioning.) The numbers under “Yes” and under “No” are the sums of the weights of each question. The DQS score for the health facility, by section and overall, is called the “quality index.” The maximum that can be obtained in each section is 10 (equivalent to 100%). For example, in Region 1, the average overall quality index related to the EIR from the health facilities visited was 66%, with the section on data collection achieving 100% (meaning that all elements evaluated were there); the lowest score, 49%, was for the section on infrastructure.

The main results of the EIR aspects of the DQS Plus from Panama are shown in Table 1 and Figure 1. The main weaknesses identified related to the limited use of the EIR to identify and track defaulters at the health facility level; obsolete software; and some limitations in the availability of human resources to support, maintain, and troubleshoot the PAI Software.

In response to the DQS Plus assessment and its recommendations, Panama procured new computers for the national EPI; conducted a national workshop with all health regions to present the results of the DQS Plus and had the regions do a data desk review; and began work to revamp and update the PAI Software itself.

### Honduras

Honduras has very high vaccination coverage levels (26), The country had a DQS in an international EPI review in 2007, and ever since then, annual DQS-like activities are conducted in different health departments. The 2015 DQS was to be a DQS Plus, given that the country has been developing and piloting an EIR that was conceived in 2009. When the DQS Plus was conducted in November 2015, 6 out of 20 health departments were using the EIR, in parallel with the official aggregated EPI information system for doses administered that was used to produce official vaccination coverage estimates.

**TABLE 1:** Selected results from DQS Plus assessments, by dimension, related to the electronic immunization registry, Panama, May 2014 and Honduras, November 2015

EIR dimension/Item in the dimension		Panama	Honduras
System scope			
	Included population	All population groups.	Children under 5 years old, though SINOVA has the capacity to record data for any person.
	Routine program, supplementary immunization activities, vaccines not included in the national immunization schedule	PAI Software allows inclusion of different vaccination strategies (health facility, vaccination campaigns, outreach modalities: school vaccination program, health workers, vaccination in businesses, farms, etc.). It also allows registering vaccines outside the national EPI schedule (for the private sector or foreigners).	SINOVA allows registering routine doses, in health facility or in outreach. Vaccination campaigns are registered as outreach vaccination activities. No possibility to record vaccines outside the national EPI schedule.
	How is the EIR to be used during outreach activities	Paper-based recording. The electronic tool itself is not used outside health facilities.	Paper-based recording. The electronic tool itself is not used outside health facilities.
	Previous cohorts (from paper or electronic systems)	No attempts at adding legacy data were made.	No attempts at adding legacy data were made.
	Vaccination history of new people as they are being added into the EIR	This is not done systematically.	This is not done or envisioned.
Normative and legal context			
	National eHealth strategy in place	At the time of the development of the PAI Software, no eHealth strategy was in place. In 2014, the national eHealth strategy and policies were being developed. At the same time, an electronic health record (EHR) project for primary health care for the Ministry of Health (MoH) (38) and the Social Security system was being implemented.	A plan for an Integrated Health Information System was available but it is outdated.
	EIR system compliance with national norms	There was no legislation or a normative framework for the use of PAI Software. Data collection and reporting was available in the 1999 national EPI Manual of Panama, but not in more recent versions.	There were no national regulations describing or regulating the EIR. The SINOVA instructions were only mentioned as part of the project procedures.
	Mandatory use of the EIR (including private and other sectors)	Mandatory for MoH public health facilities, but not for Social Security or private providers.	Mandatory in the health regions where SINOVA is being implemented. Social Security facilities do not use the SINOVA, but this possibility has been raised. SINOVA use by private providers is not planned.
	Legislation framework for data privacy and confidentiality	National Law 68 includes a prohibition on sharing data with personal identifiers through any means (physical or electronic) unless it is for epidemiological or academic research use by an authorized entity. There is no data encryption, but access to the systems is through name and user validation.	There is no national law or regulation from the MoH for the privacy and confidentiality of the data contained in SINOVA. Persons interviewed indicated that in practice the confidentiality of the data in registries is strongly guarded. There is no data encryption or user validation.
Architecture			
	Integration with other health information systems; integration with birth registration or civil registration systems; integration with other EPI information systems	PAI Software does not interoperate with any other EPI software, or with other software from the MoH, Social Security, or national Department of Vital Statistics. The data produced by PAI Software are not integrated into data from the national Health Information System.	SINOVA interoperates with the official immunization registration software used in non-SINOVA pilot regions. It does not interoperate with other information subsystems in the MoH. Although there is an agreement signed with the national Registry of Persons for the electronic exchange of data, a process to verify personal data has not yet been implemented.
	Software type	Visual FoxPro 6.0, standalone and client-server model.	Standalone model and client-server, in Visual Basic .NET 2010.
	Database type	Visual FoxPro 6.0 database.	Microsoft SQL Server 2012 database engine.
	Online–offline options	Only offline.	Only offline.
	Periodicity of data updates and database synchronization	Monthly, but monitoring not done systematically.	Monthly.
	Location of the database	The database, divided by calendar year, is stored locally in computers at the national EPI.	The databases are in a national server at the MoH Information Management unit, in servers in the project regions, and in computers in selected municipality headquarters within those regions.
	Technical specifications for computers for the system	Minimum configuration required: Pentium 4 processor Windows 98 or above, except for Windows 8 512Mb RAM 512Mb of free space in the hard drive	Minimum configuration required: CORE i3 processor or higher 4 GB RAM Memory 500 GB Hard Disk Windows 7 or higher For regional level servers: 4 core processor E3-1220 Xeon RAM memory, 4GB RAID configuration of 2 disks of 500 GB 7.2 K RPM serial ATA3 GBPS 3.5 Windows Server Standard 2012 OLP operating system with CALS to access the server For the national level server: Windows Server 2012 + 4 CALS for server access Intel Xeon Processor E5-2620-2.00GHZ RAM Memory 3 x 4 GB DDR3-1333 MHz Dual Ranked RDIMM Hard drive 1 TB 7.2 K RPM
	Inclusion of the module for short message service (SMS) or mobile health (mHealth)	No version of the PAI Software that would enable linkages with mHealth is available.	No version of SINOVA that would enable linkages with mHealth is available.
Maintenance and sustainability			
	Information management	Through a coordinated effort between the national Department of Statistics and the national EPI of Panama’s MoH, with support from the EPI of one health region.	Currently, any SINOVA adjustment depends on the original developer. However, the Technology Support Area in the MoH Information Management Unit is to assume this role.
	Plan for scale-up and capacity	PAI Software is not scalable and no plan to scale it was in place.	The current software version was designed to be scalable.
	Data security	Database backup procedures are not clearly defined, as a protocol, policy, or in any other written document.	There is no protocol, policy, or any other standard procedure for computer security or for backup copies, although a backup process is done weekly by the head of SINOVA in the Health Statistics Unit and in the regions.
	Management of software updates and improvements	PAI Software is usually only updated when new vaccines are introduced into the immunization schedule. Only one person at a regional statistics office knows how to update PAI Software. The database is updated, and an executable file is created. The file is copied onto a compact disk (CD) and sent to the regions; it is also sent as a downloadable file through a link in the MoH website. Statistics personnel at the regional level receive the CD or download the update and then send these updates to health facilities through various media. In the health facility, the executable file is copied and run. The file name is renamed to add the year of the update. At the time of the DQS Plus, May 2014, it was not possible to know whether all the facilities had the same version of PAI Software. The last plan to make improvements to PAI Software was in August 2010.	For any update, SINOVA’s developer sends the update to the Information Management Unit system manager and to the regions.
	Management of errors, users’ questions, and duplicates of records or of persons in the EIR	The person who identifies an error (usually a data entry clerk) notifies the regional level. If this level cannot resolve the problem, the regional level reports it to the region that supports PAI Software in lieu of the national EPI. The entity solving the problem depends on the nature of the error. There are no strategies to help users, such as a help desk. There are no norms or defined procedures for identification and correction of duplicate records or persons in PAI Software.	Management of incidents or users’ questions was not included in SINOVA guidelines, but a plan to do so existed. In practice, health regions and municipalities have a dedicated notebook for incident recording. This information is sent to the national level, including screenshots if needed. The technical support activities provided to the user to solve problems in the SINOVA functionality are remote and provided by the SINOVA development consultant using TeamViewer software. If there are computer operating problems, the support is given by the health region’s computer specialist. SINOVA includes validations that do not allow duplicate data to be entered. In practice, there may be duplication, such as the same person with different names, a repeated identification number, or the same person registered in different regions/municipalities. Future plans include cleaning the national database and then returning the clean database to the lower levels on a periodic basis.
	Documentation up to date	No technical documentation on the architecture or other key features of the software is available. Nevertheless, there is a user manual that is revised with each software update and distributed simultaneously.	The technical documentation on the architecture and internal operation of the software and the user manual are outdated. A clear definition of what software technical documentation (entity relationship diagrams, data dictionary, Unified Modeling Language (UML)) that needs to be made available to the MoH has not been specified.
	Financial plan for maintaining the EIR	There is no budget for maintenance, updates, or improvements to the PAI Software.	There is no budget for maintenance, updates, or improvements to the SINOVA software.
Human resources			
	Profile of data entry personnel	Profile for data entry clerks: technical or bachelor’s degree statistical level 1, 2, or 3 as per MoH organizational structure must be certified by the Health Statistical Association of Panama	There is no standardized profile for the human resources that manage SINOVA, but whenever there is new staff to do data entry, they are trained in vaccination schedules and other relevant EPI information.
	Profile of person responsible for validating the data and monitoring potential record duplication	No defined official profile for the person doing data validation.	No defined official profile for the person doing data validation, but this task has been assigned to the head of the Information Management Area and the person in charge of the EPI in the health regions.
	Profile of software developers; profile of trainers; profile of people in charge of maintaining hardware and telecommunication infrastructure; profile of database administrator	Profiles required not described. Available personnel lacked minimal technical skills needed for developing and administering a database, for training on the use of the software, or for maintaining the hardware and telecommunications used by PAI Software. No defined official profile for tech support.	No technical profile defined for database administration personnel, software development, and/or maintenance and training in the use of software, nor for technicians for hardware and telecommunications maintenance.
	Help desk	No help desk strategies for PAI Software users.	No help desk strategies for SINOVA users, but there are multidisciplinary coordinating teams, at national and regional levels.
Modules included in the system			
	Immunization registry	PAI Software has an immunization registry module and a module for aggregated data on doses administered.	SINOVA has an immunization registry module only.
	Logistics and supply chain management	No	No
	Cold chain inventory	No	No
	Surveillance of adverse events following immunization	No	No
	Vaccine-preventable disease surveillance	No	No
	Training module	No	No
	Other modules	No	No
	EIR functionalities		
	Following individual vaccination schedules	No	Yes
	Coverage monitoring	No	Yes
	By age	Yes	Yes
	By condition (pregnancy, chronic diseases, etc.)	Yes	No
	By geographical area	Yes	Yes
	By ethnicity/minority group	No	Yes
	By health facility vaccinating	Yes	Yes
	By vaccinator	No	No
	By health system affiliation	Yes	No
	Vaccine lot number monitoring	Yes	No
	Recalls/reminders (automated generation)	No	No
	Management reports	Yes	Yes
	Ad hoc reports	Yes	No
	Predefined reports	Yes	Yes
	Validation modules	Yes	No
	Duplicate record management	No	No
	Map generation	No	No
	Georeferenced data	No	No
	Access for external users	No	No
	Communication between EPI and EIR users	No	No
	Information dissemination	No	No
	Clinical decision support on immunization	No	No

***Source:*** DQS Plus results from Panama in 2014 and Honduras in 2015.

#### The EIR system in Honduras.

In 2009, the Ministry of Health of Honduras decided to embark on a joint project between its Expanded Program on Immunization and its Health Statistics Department to develop SINOVA (from the Spanish *Sistema Nominal de Vacunación, *or Name-based Vaccination [Information] System), an immunization information system for vaccine doses administered. SINOVA includes a module for aggregated vaccination data and one EIR. The system was developed in Visual Basic .NET 2010, an object-oriented programming language. The system has an SQL database, Server 2008-2012, as its database management system, and it uses a three-layer model (data access, business logic, and user interface). The client/server application allows sharing the database with various workstations in the same network and connecting to the SINOVA database on the same or different servers.

SINOVA includes personal data for children under 5, with the registration number (unique to SINOVA), complete child’s name, date of birth, sex, place of birth, address, ethnicity, and name of the mother, as well as information on the vaccines administered (type, date of administration, etc.) and facility vaccinating.

##### Data flow.

In regions using SINOVA, vaccinators in all health facilities record (on a paper form named SINOVA-1 that allows recording multiple children on a single page) each child’s information and information on each vaccine dose administered, whether at the health facility or through outreach.

At the end of the day, the EPI nurse manually compiles the vaccination data from SINOVA-1, by day and vaccine type and dose, into an aggregated form named SINOVA-2. The SINOVA-2 includes data on the health facility, the municipality, and the department, as well as the name and signature of the person responsible for EPI. Once a month, all SINOVA-1 paper registers are submitted to the municipal level, where they are manually checked for completeness and data consistency. Then, within the first seven days of each month, all forms are sent by the municipalities to the health regions. Data entry in the SINOVA software takes place at the regional level in most areas, and at the municipal or network level (municipalities administratively grouped) in a few areas. SINOVA-1 registers entered into the database are then returned to the health facilities. For EIR data, on the 10^th^ of each month, the health regions using SINOVA are to send the database to the national level, usually by email.

##### Main results.

**FIGURE 2:**
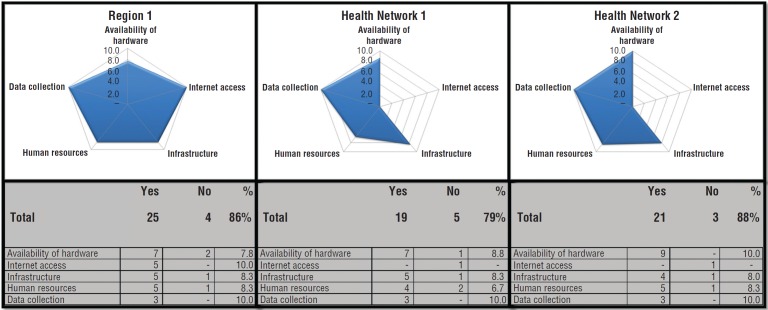
Sample DQS Plus results, by region or health network, Honduras, 2015^a^

***Source:* DQS Plus results from Honduras in 2015.**

^a^ The term “health network” refers to the lowest administrative level charged with managing the immunization program. In the subfigures, each item (availability of hardware, Internet access, infrastructure, human resources, and data collection) represents a section included in the DQS Plus data quality questionnaire/checklist. The numbers under “Yes” and under “No” are the sum of the weights of each question. The score found in the DQS, by section and overall, is called the “quality index.” The maximum that can be obtained in each section is 10 (equivalent to 100%). For example, in Region 1, the overall quality index related to the EIR was 86%, with the sections on Internet connection and on data collection achieving 100% (meaning that all elements evaluated were there). The lowest Region 1 score, 78%, was for the section on availability of hardware.

The main results of the EIR aspects of the DQS Plus from Honduras are shown in Table 1 and Figure 2. The main weakness identified that was related to the use of EIR was the limited infrastructure: the municipal levels evaluated did not have Internet connectivity. Additionally, there were limited human resources to enter data and to support, maintain, and troubleshoot SINOVA.

The main recommendation from this DQS Plus was that SINOVA must be used at the health facility level to support vaccination activities. Following the DQS Plus, Honduras has continued the SINOVA implementation processes in other regions, in addition to the ones using SINOVA at the time of the DQS Plus; has improved the reports module; and has continued improving and updating the system. In addition, in 2016 the national EPI conducted a training workshop to discuss data quality activities and to share good practices for the use of SINOVA.

### Issues common to both Panama and Honduras

In both Panama and Honduras, the EIR does not yet meet all the working criteria set by PAHO for an “ideal” EIR. Both EIRs sought to include all persons as early as possible, when Bacillus Calmette–Guérin (BCG), a vaccine against severe forms of tuberculosis, is administered at birth, or in the first days following birth. However, no EIR had linkages to birth registration. While both EIRs included a field for a unique ID, birth registration is delayed in many cases in the two countries, resulting in the use of a temporary ID that may lead to duplication. No standard de-duplication protocols were available in either country.

Regarding the information included in the EIRs and some of their functionalities, data about each person were included, such as area of residence, the vaccines given, dates, and the provider. Also, both EIRs had functionalities that allowed aggregating data on vaccine doses administered by geographical area. Nevertheless, two key features of a well-constructed EIR that are linked to the ability to help increase vaccination coverage were limited in both countries: 1) using the EIR for provider assessment and feedback and 2) facilitating timely follow-up with defaulters. These limitations may be explained by issues related to infrastructure and connectivity. For example, data entry into the EIR was not done by the vaccinators themselves, but by statistics staff, usually at the health facility level in Panama and by EPI staff, but in administrative units, in Honduras. Furthermore, an up-to-date database with the EIR was not always available at the health facility level. Protocols for data security and protection of patient confidentiality needed improvement in both countries, but particularly in Panama. The most relevant recommendations for improvement were related to enhancing the use of the EIR to follow up with defaulters; managing the databases; and adding functionalities and improvements to the informatics tools themselves.

In both Honduras and Panama, improvement activities are being implemented. However, the Immunization team at PAHO hasn’t measured the progress of those improvements.

## DISCUSSION

As part of the work aimed at improving immunization data quality and use, LAC countries continue developing and implementing EIRs (3, 10, 27). Nevertheless, systematic methods and tools to assess EIRs in LMICs, and even in high-income countries, are lacking. PAHO sought a practical approach to EIR assessment, by adding an EIR module to the regional version of the DQS and thus creating DQS Plus. The decision to develop an EIR assessment module that could be integrated with the DQS was based on the fact that the DQS is a well-established tool in LAC.

To our knowledge, this is the first methodology proposed to assess not only data produced by an EIR, but also elements related to the EIR itself, for use in LMICs. The experiences in Panama and Honduras illustrate that the DQS Plus approach proved practical and easy to implement. The DQS Plus did not add additional days to the assessment, and it provided practical and actionable recommendations, which in both countries led to actions aimed at addressing the issues identified in the DQS Plus. Furthermore, including persons from the areas of immunization, statistics, and informatics in the teams facilitated implementing the multidimensional DQS Plus approach and the development of the improvement plans, given that activities to improve an EIR are often to be implemented by a multidisciplinary team.

An important aspect of the DQS Plus was the attempt at assessing user satisfaction, by adding a separate set of questions for EIR users of the DQS questionnaires. A recent study about electronic health records (EHRs) in hospitals in Ethiopia found that among all the constructs studied, user satisfaction showed the strongest effect on perceived net benefit among health professionals (28). In both Panama and Honduras, the interviewed users were satisfied with their EIR. This was especially true for nurses in Panama, for whom PAI Software facilitated the monthly aggregation of data and reporting that they have always had to do for EPI.

The DQS Plus is subject to important limitations. First, the DQS Plus does not use a sampling method that can provide results generalizable to the entire country. This indicates the need to use caution when interpreting the qualitative results displayed as radar graphs and the findings from the user satisfaction questionnaires. Second, there were no attempts at understanding the level of computer literacy among EIR users, which for EHRs has been found to be an important factor for user satisfaction and EMR use (28). Third, and perhaps the most important limitation of the DQS Plus, is that given there are no commonly accepted EIR standards against which one could compare the findings from an EIR assessment, benchmarking is not yet possible. Therefore, most of those findings can only be considered descriptive.

Benchmarking, that is, establishing standards of excellence and comparing indicators to those standards, has been amply used in health care as a tool for continued quality improvement, where indicators are monitored and compared to apply best practices and to create a spirit of healthy competition (29, 30). In the United States of America, EIR goals have been set, and the U.S. CDC annually surveys jurisdictions about their immunization information systems through an annual self-administered questionnaire focusing on four priority areas: 1) data completeness; 2) bidirectional exchange of data with EHR systems; 3) clinical decision support for immunizations; and 4) the ability to generate childhood vaccination coverage estimates (31).

EIRs in LMICs are relatively new, and experiences are still being collected and shared (3, 4). It is not yet clear what factors relate to the successful implementation of an EIR; such information could make it possible to make predictions about and conduct measurements on an EIR as it is being designed, developed, and piloted. How much can be learned from the world of information systems in general (and EHRs in particular) and then applied to EIRs in LMICs is an area to be further explored (32-42). PAHO’s Immunization Unit has been working with the PAHO eHealth and EHR teams, as well as with the Expanded Program on Immunization team at the World Health Organization headquarters, the American Immunization Registry Association, the Better Immunization Data initiative, and the U.S. CDC Global Immunization Division to improve the DQS Plus.

A DQS Plus done in Grenada’s EIR in mid-2018 added new dimensions to the assessment methodology, related mainly to EIR costs, maintenance, and sustainability planning. The version of the DQS Plus used in Grenada followed the framework recently proposed by PAHO’s EIR module, which was published in February 2018 (43). The Grenada DQS Plus also included a structured module to assess the EIR data. This EIR data assessment was done by selecting a random sample of data from the EIR database to determine the existence of potential duplicate records or users, and to assess data completeness and consistency. The evaluation also compared records in the EIR being assessed against other databases or coverage estimates, as has been done in Australia, Uruguay, and some provinces of China (4, 15, 19, 44).

Better assessing EIRs will help develop standards and better guidance for EIRs in LMICs and help predict the possible level of success for a new EIR before countries embark on costly development and implementation. The ultimate goal of EIRs is to help produce quality vaccination data so that all levels of an immunization program, from the delivery of the vaccinations to the management of the national immunization program, can work more effectively and efficiently in reaching and vaccinating people, and thus reduce and even eliminate vaccine-preventable diseases.

## Author contributions.

MCD-H envisioned the DQS Plus. MCD-H, MPC, and MV-G envisaged the article. MCD-H, MPC, DP, and DM led the DQS Plus in Panama. MPC, IBM-A, OG, and MV-G led the DQS Plus in Honduras. All the authors reviewed and approved the final version of this article.
